# Late History of Cattle Breeds in Central Europe in Light of Genetic and Archaeogenetic Sources—Overview, Thoughts, and Perspectives

**DOI:** 10.3390/ani14040645

**Published:** 2024-02-17

**Authors:** Vojtěch Janák, Karel Novák, René Kyselý

**Affiliations:** 1 Institute of Archaeology of the Czech Academy of Sciences, Prague, Letenská 4, 118 00 Praha, Czech Republic; 2 Department of Genetics and Breeding, Institute of Animal Science, Přátelství 815, 104 00 Praha, Czech Republic; novak.karel@vuzv.cz; 3 Department of Archaeology, Faculty of Arts, Charles University, Nám. Jana Palacha 2, 116 38 Praha, Czech Republic

**Keywords:** historical cattle, Czech Red cattle, aurochs, sexual dimorphism, osteometry, hornlessness, archaic DNA

## Abstract

**Simple Summary:**

Although Europe was not a primary centre of cattle domestication, the cattle expansion from the Middle East and subsequent development created a complex pattern of cattle breed diversity. Many isolated populations of local historical breeds still carry the message about the physical and genetic traits of ancient populations. Historical cattle diversity is currently at the intersection of two leading directions of genetic research. Firstly, it is archaeogenetics attempting to recover and interpret the preserved genetic information directly from archaeological finds. Secondly, it is collecting genomic information about the previous development of extant populations. The present paper aims to place selected archaeogenetic, genetic, and genomic findings in the picture of cattle history in Central Europe, as suggested by archaeozoological and historical records. The importance, actuality, and effectiveness of combining different approaches to each archaeological find, such as morphological characterization, interpretation of the historical context, and molecular data, are stressed.

**Abstract:**

Although Europe was not a primary centre of cattle domestication, its expansion from the Middle East and subsequent development created a complex pattern of cattle breed diversity. Many isolated populations of local historical breeds still carry the message about the physical and genetic traits of ancient populations. Since the way of life of human communities starting from the eleventh millennium BP was strongly determined by livestock husbandry, the knowledge of cattle diversity through the ages is helpful in the interpretation of many archaeological findings. Historical cattle diversity is currently at the intersection of two leading directions of genetic research. Firstly, it is archaeogenetics attempting to recover and interpret the preserved genetic information directly from archaeological finds. The advanced archaeogenetic approaches meet with the population genomics of extant cattle populations. The immense amount of genetic information collected from living cattle, due to its key economic role, allows for reconstructing the genetic profiles of the ancient populations backwards. The present paper aims to place selected archaeogenetic, genetic, and genomic findings in the picture of cattle history in Central Europe, as suggested by archaeozoological and historical records. Perspectives of the methodical connection between the genetic approaches and the approaches of traditional archaeozoology, such as osteomorphology and osteometry, are discussed. The importance, actuality, and effectiveness of combining different approaches to each archaeological find, such as morphological characterization, interpretation of the historical context, and molecular data, are stressed.

## 1. Recapitulation of the Domestic Cattle Origin—Present State of Knowledge

### 1.1. Impact of the Question of Cattle Evolution

With the advent of farming, the domestication of cattle was one of the most important events in human transition from hunter-gatherer to the new agricultural lifestyle. Throughout subsequent millennia, cattle domestication played a major role in the subsistence and economy of a large part of the world, including central Europe, and in modern times cattle are one of the most important domestic animals. It was one of key components of neolithization in Europe. Cattle played a decisive role in later economic transitions involving animal power utilization in terms of the so called “Secondary products revolution”, formulated by A. Sherratt [1,2], in Central Europe probably in the 4th millennium BC. As such, its history and colonization of the wide area in which it is found today have been subject to intensive research by archaeozoologists, and more recently also by archaeogeneticists and population geneticists worldwide. The archaeological findings can be interpreted in light of the genetic data and vice versa.

### 1.2. Two Centres of Origin

There are two variants of domestic cattle—taurine (*Bos taurus*) and zebu (*Bos indicus*)—which arose from distinct aurochs subspecies (*Bos primigenius primigenius* and *Bos primigenius namadicus*). The two subspecies’ separation took place about 300,000 years ago [3]. The latter is, however, adapted to warmer, subtropic, and tropic conditions, and has never been raised in Europe in a significant way, while the European breeds belong to the taurine lineage. They are identified genetically by the presence of the T macro-haplogroup, i.e., a characteristic set of single-nucleotide polymorphisms in mitochondrial DNA (mtDNA). On the other side, zebuine cattle are characterized by the I1 and I2 zebuine haplogroups [4].

### 1.3. Historical Development of European Cattle Breeds

The question of the origin of European cattle has long been approached with two main working versions, namely a local origin of cattle through parallel domestication events in multiple regions, or a singular origin with subsequent spread throughout the continent. With increasing development and use of genetic methods, this question is all but answered today. Taurine cattle were first domesticated in the Fertile crescent some 10,500 years ago, certainly by the pre-pottery Neolithic B stage [5]; the combination of results of archaeozoological and genetic research suggests that this probably occurred in the upper or middle Euphrates region [5,6,7,8]. The original founding population is thought to have been very small, perhaps comprising only 80 females [9]. Importantly, it is clear that taurine cattle form a single group, confirming that all European cattle descended from this domestication event [10,11]. Conversely, no other independent domestication of *Bos primigenius primigenius* has been discovered, although this has only been directly tested at one site up to now [12].

After domestication, cattle gradually spread westwards with the advancing of the Neolithic. The North African population formed a distinct subgroup that is now genetically characterized by the prevalence of the T3 haplogroup of mtDNA, and by only limited contact with Europe [13]. Furthermore, the introgression of local subspecies of aurochs (*Bos primigenius mauretanicus*, *syn. Bos primigenius africanus*, *Bos primigenius opisthonomous*) is assumed. A recent communication [14], however, re-opened the question of an independent third domestication centre in North Africa, as based on the archaeological material from the Althiburos site in Tunisia.

In Europe itself, cattle were probably introduced via the Aegean around 7000 BCE or later, and then spread along two routes as a component of the so-called Neolithic package. One branch went along the Mediterranean coast, while the other simultaneously crossed the Balkans and advanced via the Danube valley to Central Europe. Cattle first appeared there in the middle of the 6th millennium BCE [10]. A newly described T6 haplogroup exists both in present day Shorthorn Rhodopean cattle, and zooarchaeological samples suggesting an antiquity of Balkan brachicerous cattle and a long continuity of local breeding [15]. In the Central European region of Bohemia, domestic cattle have been documented since about 7700 BP [16,17]. It was a gradual process consisting of several successive founder events, inevitably leading to the decrease in genetic diversity with increasing distance from the Near Eastern area of origin [10].

### 1.4. Cattle Diversification Process

The general trends in the domestication process in an array of species, including cattle, as illustrated on the findings in the Western European region of the Basque Country have been summarized by Altuna and Mariezkurrena [18]. A very important genetic event was undoubtedly the contact between both aforementioned routes of neolithization in Europe. After contact in northern France and other areas, these two cattle populations could interbreed. The presence of domestic cattle of a very small size, typical for western and south-western European regions, was observed in southern Germany [19,20] and is presumed to spread as far as to the northern Pre-Alps [21,22], but this form seems not to be found further eastwards or in the north.. The influence of a smaller ‘western’ breed has not been described yet and is not expected to be present within the Czech lands [23]. Further on, the size of cattle decreased, which fact has been reported many times from many European regions. Examples include the interface between the Bronze and Iron Age in the Arenaza cave explorations in the Basque Country [24] in Western Europe and further north-east the time span from the Neolithic to the Middle Ages in the Czech lands [23].

In addition to the above-described broad genetic processes and events, small-scale genetic processes have undoubtedly occurred across time and space in Europe, giving rise to distinct local breeds. These phylogeographic nuances remain to be discovered. If we want to reconstruct the history of cattle, the best course of action would be to focus on later periods, such as the Middle and Modern Ages. In these periods, we can search for the direct roots of recent breeds. The detailed knowledge of genetic constitution of modern breeds, brought about by their economic role, is undoubtedly an ideal starting point for reconstructing the details of the late history of the mentioned periods.

As far as Middle and Modern Age history are concerned, we must take into account mobility, especially the intensity of trade. In the Czech lands, for example, the continuous and large-scale imports from the Kingdom of Hungary were established in the middle of the 14th century [25]; a significant item was cattle (oxen). This trade was paralyzed after the conquest of Hungary by Ottoman Turks in 1541, but the cattle exports from Upper Hungary (Slovakia) via Moravian markets to Prague and other destinations persisted in the 16th century, e.g., 1500 oxen were driven to Prague for direct local exploitation in 1597 [25]. If we would include osteological material after the mid-14th century periods in archaeogenetic research, we must be aware of the presence of imported cattle remains in settlement waste. We believe that the chance of detecting local breeds is greater in rustical contexts, and the chance of detecting imports is greater in centres such as Prague or other towns. The impact of these imported cattle on the gene pool of local breeds remains an open question. Generally, it is assumed that the imported cattle were determined to be slaughtered and consumed, and in such case there would be no or small genetic influence on local population. Moreover, the general focus on oxen (castrates) excludes their incorporation in breeding. So, a more or less independent development of a local (Bohemian) breed without the admixture of foreign influences can be expected. Anyway, a guiding indicator might be the prevailing combination of mtDNA and Y chromosome haplotypes: while the former illustrate more stable female population, the latter data correspond to the possible long-distance trade with bulls.

A curious contribution to the reconstruction of the evolution of cattle is a comparatively recent analysis of the mtDNA haplotype in the Tyrolean Iceman shoelace, made of bovine leather [26]. The T3 haplotype recovered from the artefact corresponded to the cattle population from this era (5300 years BP), and also to the extant population of the Central European breed Czech Red cattle (unpublished). Another artefact from which DNA has successfully been extracted and analysed is a cattle rib fragment containing Germanic runes from Lány, Czech Republic [27]; the authors ascribed the find to European domestic cattle. Obviously, even artefacts of materials of animal origin might contribute to the picture of cattle development in the region.

## 2. Possible Crossbreeding and Aurochs Introgression

### 2.1. Evidence for Crossbreeding with Aurochs

One of the ongoing debates regarding the history of cattle is the influence of native European wild cattle, i.e., the aurochs of the subspecies *Bos primigenius primigenius*. This animal has been present in Central Europe since the Pleistocene, and this area was the last refugium of the species until the last individual died in 1627 in Jaktorow near Warsaw, Poland. The general prehistory and history of aurochs in Central Europe has been reviewed by several authors [28,29,30,31,32,33,34]. Therefore, it must be considered that domestic and wild cattle coexisted here for approximately 7000 years.

Genetically, there is no significant reproductive barrier to successful crossbreeding between wild and domestic cattle, as they represent essentially two forms of the same species. The question is: Did such crossbreeding take place? If so, then when, where, and at which frequency? Before the emergence of archaeogenetics in recent decades, several alternative hypotheses have been considered, including local European domestication suggested, for example, by S. Bökönyi [35,36,37]. This hypothesis is based on a large amount of archaeo-osteological material from Hungary dated to the Herpály, Theiss, and Lengyel cultures. However, this possibility was doubted even before the genetic evidence emerged, e.g., [38]. Later on, as discussed above, the genetic analyses demonstrated conclusively that European domestic cattle do not represent a local domestication event, or even multiple events, but the entire population shares a Near Eastern origin.

The common origin, however, does not rule out local interbreeding on some scale. In fact, evidence points towards this scenario being the case. Some, although low, contribution of European aurochs is detected in recent breeds. Both the morphometric evidence and mtDNA data support this hypothesis. The aurochs trait introgression has been reported mainly for findings in Italy [4,7,13,39,40,41,42]. Consistent with this, a recent study has found surviving aurochs mtDNA lineages in modern taurine cattle, namely in the alpine Murbodner breed in Austria. This again confirms that interbreeding happened and that it had a traceable impact on the local cattle population [43].

Later, several instances of probable domestic cattle bearing aurochs DNA have been discovered also based on archaeogenetic data. For example, an instance of morphologically domestic cattle carrying aurochs mtDNA is known from the Bronze Age site in northern Spain, and so assumed to be a descendant of an interbreeding event [44]. In Central Europe, an animal corresponding in size to a domestic cow was discovered to have aurochs DNA at the Swiss Neolithic site of Twann [45]. The magnitude of these events and scenarios is difficult to judge, but in the case of the latter, interesting speculation is possible. Based on general ecological data for the site, wild aurochsen have probably largely relocated to different sites, and the chance of encountering domestic cattle was therefore unlikely [45].

Another case is a large animal in the Early Medieval context of Vyšehrad (Prague, Czech Republic), which was suspected to belong to aurochs by morphological traits. However, archaeogenetic analysis revealed the T (domestic) haplogroup. Since, in the course of the well-documented size-decreasing domestication trend, Early Medieval cattle reached the smallest body size and very small horns (for the Czech lands, see [16,24]), the large-horn animal was classified as a suspected domestic-wild crossbred [46]. The mtDNA haplotype corresponding to the domestic cattle haplotype T3 suggests the possibility of aurochs male–domestic-female interbreeding, and possibly illustrates its relative geographic and temporal spread.

**Figure 1:**
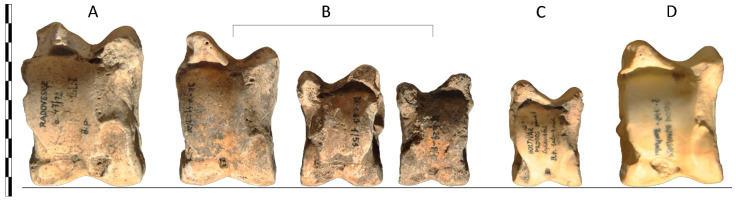
Size variability of the *Bos* talus (ankle bone) compared to the talus of wisent. The line of ankle bones represents, from the left, wild cattle (aurochs, *Bos primigenius*) from the Roman site Radovesice (**A**); cattle individuals ranging in size from aurochs to domestic cattle from Eneolithic hillforth Kutná Hora-Denemark (**B**); domestic cattle from Prague-Hostivař dated to the Iron Age (**C**); and recent female wisent (*Bison bonasus*) (**D**). The bones are analysed and photodocumented in the archaeozoological laboratory of the Institute of Archaeology of the Czech Academy of Sciences in Prague (Photo: R. Kyselý, Prague, 2023).

Until now, the size and morphology of the bones has been the principal feature used in distinguishing aurochs from domestic cattle, e.g., [47,48]. Individuals of intermediate size between aurochs and domestic cattle are quite common in Neolithic–Eneolithic periods, but their interpretation is questionable. Some authors, based on various morphological and other non-archaeogenetic arguments, concluded that the most probable interpretation is crossbreeding. In parallel to the works by Bökönyi [35,36,37,49,50,51], Lasota-Moskalewska [49], Müller [50], and Döhle [51], it was suggested and deeply discussed in works based on the osteological materials from Bohemia [24,33]. However, one must be careful about such interpretations. The observed continuous size distribution can be a result of various phenomena [24,38]. The occurrence of unusually large domestic cattle individuals or small aurochs individuals cannot be fully ruled out [12], especially when sexual dimorphism present in both types is taken into account. As a morphometric assessment of the sex of the specimen usually requires bones to be preserved whole, the fragmentariness further complicates identification. Furthermore, the results can be affected by sample size, since smaller collections can result in the separation of finds into more discrete clusters [52]. Size variability in bovids is illustrated in Figure 1. The presented material originating from Kutná Hora-Denemark is one of the assemblages suitable for the study of intermediate forms, possibly crossbreds [24,33].

### 2.2. Conclusions from Y Chromosome Variability

In contrast to the studies based on mtDNA and those documenting maternal lines, studies focusing on autosomal or even Y-chromosome DNA analysis are less common. Still, this direction was attempted relatively early. The first results showed two haplogroups within European breeds, called Y1 and Y2 [53]. Their distribution showed a relatively clear line, with Y1 dominating in Northern and Western Europe, and Y2 in southern regions and Anatolia. This pattern has been interpreted as crossbreeding between domestic cattle and aurochs. More frequent crossbreeding of wild male × domestic female than vice versa is assumed, especially in northern areas of Europe, if European aurochs bulls carry the Y1 haplogroup [53].

However, these results were not supported by the subsequent work by Bollongino et al. [40] who investigated the issue further with the inclusion of ancient samples for sites with a wider geographic range. Their results were markedly different. Both haplogroups were detected, but samples from local aurochs only yielded the Y2 variant, effectively refuting the preceding interpretation. As for Central Europe, both haplogroups were detected [40]. This does not exclude the possibility of some of the Y2 haplogroup present in modern breeds coming from the aurochs introgression. However, no clear indicator is available, and the extent of these crosses remains uncertain [40,54]. In any case, the combination of wild male and domestic female in prehistorical mating seems to be easier and therefore more likely than the opposite scenario, which would assume attracting wild females by domestic males with lower fitness and probably disadvantaged due to their smaller body frame. The mating could have been intentionally arranged by farmers, or could have occurred by chance [24,33]. Accidental mating between wild males and domestic females has been reported in other species, namely the yak (*Bos mutus*). These instances, however, usually see the females joining wild herds, and the resulting hybrids are noted as being difficult to domesticate [55]. Such behaviour does not automatically translate between species, but can be considered as a possibility which would result in unintentional mating leaving a limited admixture in the domestic cattle gene pool. In such a scenario, mitochondrial DNA analysis would not be able to detect the contribution of male aurochs to the domestic cattle population. However, such crossbreeding could be detected by the Y chromosome analysis instead.

## 3. Morphological and Health Traits for Medieval Cattle Evolution

In any case, the morphological features such as hornlessness, horn type, coat colour, as well as resistance against certain illnesses are questions which could be studied and solved by archaeogenetic research based on bone finds. Some morphological features such as hornlessness can be identified and studied mutually with osteological observations, but also in the situation when relevant (cranial) anatomy did not persist in archaeological materials. In any case, archaic DNA (aDNA) studies can help to clarify the origins of this phenomenon. This is expected to be accomplished by targeted resequencing and genotyping for allelic variants in additional genes of key importance for local adaptation and exploitation in agriculture, as exemplified by genes for polledness and related states (*P*/*p*, *Sc*/*Sn*), and genes for coat colour like *KIT* and *MC1R* (melanocortin 1 receptor) [56,57,58].

It is also generally accepted that local breeds are a result of adaptation to specific endemic disease challenges. This is relevant for archaeogenetics, since the information from findings illustrating pathological changes can document this process. On the other hand, the historical changes in the immune system can be illustrated by ancient DNA structures. In addition, historical breeds can be a source of diversity for breeding for resistance in modern breeds [59]. However, this line of research received less attention than morphological or coat colour traits. Variability in the bovine immune gene *TLR4* has already been reported for the finds on the island Öland from the Early Middle Ages [60], and been associated with resistance to cattle plague. On the other hand, immune gene variability has been documented for many historical breeds, with the intention of finding the separation points from the modern breeds. However, these data can be used to search for the match with archaeogenetics materials. For example, the MHC polymorphism in the historical breed Czech Red cattle has been documented as early as in 1996 [61]. The polymorphism in the members of toll-signalling pathway for the innate immunity system of has been documented for this breed as well [62], which links the possibility of studying the medieval breeds in the region for this feature and the extant Czech Red Pied cattle.

## 4. Modern Breeds of the Region and Their Links to History

In today’s Europe, commercial cattle belong to a few selected breeds that became dominant in the second half of the 20th century and have been heavily influenced by intense breeding for economic efficiency, as facilitated by modern technologies such as artificial insemination. These include the most widespread Holstein-Friesian cattle, originally from northern Germany and Denmark, Charolais from central France, or Simmental from Switzerland [63]. Populations of these breeds often number in millions [64]. DNA polymorphism can be used to detect the geographical movement of animals as an indicator of long-distance trade, the migration of human populations, and military expeditions [65].

However, the rapid genetic development of these cattle breeds and the increase in commercial gain brought significant drawbacks. Firstly, inbreeding has massively increased in these populations. This process can be characterized by the so-called effective population size, i.e., the least population comprising all variability. For example, in a total population of almost 1,900,000 Holstein-Friesian cattle in Denmark, the effective population size was comprised of only 49 animals [66]. A second problem arising from the switch to several highly productive breeds that corresponds to the focus of the present article is that local breeds, unable to compete in terms of economic productivity, were driven to marginality and, in some cases, to extinction [64]. Still, some local breeds managed to endure, and in recent decades, there is a growing awareness of the value these breeds provide as a reservoir of genetic diversity. Among others, the resistance of local breeds against some illnesses, epidemics, and climatic deviations is considered. Moreover, the genetic information from the extant populations can efficiently complement fossil DNA data.

### 4.1. Red Mountain Cattle Cluster

**Figure 2:**
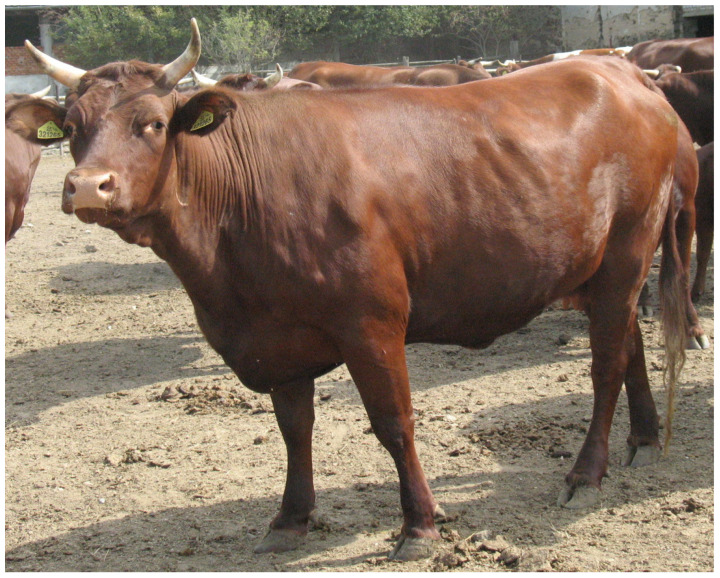
The characteristic animal of the current herd of Czech Red cattle (Photo: K. Novák, Lány, 2018).

One group of breeds present in central Europe is the so-called Red Mountain cattle. These breeds can be found in higher altitude regions through Germany, Poland, Slovakia, and the Czech Republic [67]. Originally, they were used universally as milk, meat, and draught animals. Currently, there exist programs focusing on preserving and invigorating these populations; however, they all experienced significant bottleneck events in the past. At the beginning of the 21st century, there were about 1000 cows of Polish Red cattle present in Poland [68]. In the former Czechoslovakia, the population of the so called Czech Red cattle (Figure 2) decreased to 15 individuals in 1987. Consequently, the diversity of the Czech Red herd is now reaching the effective size of only 11 [69].

Another member of the Red Mountain cattle cluster, the German Red Mountain cattle, also Harzer Rotvieh, Harz Mountain cattle, or Rotes Höhenvieh, originated by merging several local forms from different mountain regions [67].

Salers cattle, characteristic of the mountainous regions of France, is also often associated with this group of breeds. However, the attempts to associate Red Mountain cattle with bull paintings in Massif Central must be rejected. This possibility is excluded by simple dating of these paintings to 16,500 years BP (https://salerscanada.com/the-salers-breed/history/; accessed on 15 February 2024). The match of the external traits seems to be simply a coincidence.

Labelled as local, the current state and appearance of these breeds represent development during the last several centuries. In outward appearance, they are of medium size with red or yellow-red colouring [69]. Generally, it can be said that the so-called red cattle form a group closely related to each other. The exact relationships between the breeds differ between different approaches. The microsatellite assay indicates that the Czech and Polish Red cattle are the close populations to German Red Mountain cattle [69]. Another study disagrees, as it finds the Czech Red cattle sharing most similarities with the Czech Simmental breed [70]. However, as noted in the latter study, this comes as a little surprise, because the Czech Simmental developed in the 19th century partially from the Czech Red cattle. Also related to the Central European group are the Baltic Red breeds, as seen by the relationship of Polish and Lithuanian Red cattle [71].

There is no doubt that the historical cattle breed Czech Red cattle can be a unique source of information about the history of the cattle population in the Labe and Morava river basins. The breed is characterized by low effectiveness in parallel to prominent health traits. In the Czech cattle breeding literature, the ancient origin is often speculatively associated with the Celtic shorthorn breed or with the admixture of *Bos primigenius.* The purposeful conservation efforts can be dated back to 1921; however, the breed became almost extinct in the 1980s. The current population has been restored from 1 bull (with 50% admixture) and 14 cows in 1987. The current herd consists of approx. 200 animals which are included into the Program of Conservation and Utilization of Farm Animal Genetic Resources in the Czech Republic (https://vuzv.cz/en/genetic-resources/; accessed on 15. February 2024). On the other hand, it is generally recognized that the population contains admixture from a number of breeds, namely Polish Red, Angler, German Mountain Red cattle, Czech Simmental cattle, Ayrshire, Red Holstein, and Piedmont cattle. This situation is apparently the result of crossbreeding with the goal of improving the productivity of the local breed. These efforts also affected the genetic background of the Czech Red Pied cattle (of the Simmental breed group) by intercrossing with the modern Holstein Friesian population [69,70]. In particular, the diversity of mitochondrial DNA for the existing populations of the Czech Red and Czech Red Pied cattle is documented, and indicates admixture from south-eastern European regions [72].

However, the introgression of the modern gene forms into the historical gene pool of the Czech Red cattle is not a unidirectional process. The genetic background of the modern Czech Red Pied cattle breed might contain a significant proportion of gene variants from the preceding cattle breeds in this territory. The breed is a member of the Simmental group and was formed by imports of true Simmentals during the 19th century [73]. The local cattle population was absorbed by introgression; however, the presence of blocks of adaptation genes inherited from the previous local breeds is anticipated. This hybrid structure was probably conserved by non-intentional selection for adaptation traits. Consequently, the original type of this breed is conserved in the population of 80 animals also in the program of genetic resources since 2010, with a population structure that reflects the state at the end of the 1990s.

These works also bring to the forefront the fact that geographic and political factors played a significant role in the history of cattle breeding. The authors point to uniform breeding practices in former Czechoslovakia as a cause of the decline of the historical breeds [69]. Similar practices are reported from another population of Red cattle from Lithuania. Pedigree analysis of a group of Lithuanian red cows discovered 14 different breeds as ancestors of the current sample [74].

Despite this recent crossbreeding, it is highly likely that these breeds represent local and autochthonous population of cattle in their respective areas. The homogeneity of the group is further supported by the fact that most of them belong to the Y chromosome Y1 haplogroup from Bohemia to Baltic countries, and even Ukraine [54].

### 4.2. Podolian Cattle

The so-called Podolian group is a collection of breeds mainly from eastern and southeastern Europe which share a common origin believed to be in Podolia—a historic region located in the west-central and south-western parts of Ukraine—and in north-eastern Moldova. This group is regarded as ancient, and is notable for being closer in appearance to its wild ancestors than other European cattle in such traits as having generally longer horns and more pronounced sexual dimorphism [3,75]. Its origin and history are still a topic of research, with only recently appearing studies tackling the question [3,76].

Mitochondrial DNA analysis suggests two distinct subgroups of Podolian cattle, one roughly located in eastern Europe and the other from even further to the east, presumably incorporating the Turkish Grey as well as some southern Italian breeds. It has therefore been speculated that the first group may have spread through its current range during the migration period [75]. As has been noted above, one must, however, keep in mind that the breeds in their current form developed over the course of the last three or four hundred years.

It is difficult to assess the genetic impact that this mass migration of Podolian cattle had on more western breeds. Several Italian Podolian breeds have been included in studies of ancestry and admixture. In general, it was revealed that the studied breeds have a rather complex genetic composition and origin [76], including an indicine and African admixture [76,77]. Originally thought to originate in the time of cattle domestication, the indicine component was recently discovered to be of a more recent origin. A sharp influx of indicine cattle into the local taurine populations around 4000 years ago was detected in a DNA study of Near East cattle remains [11]. As such, it has reshaped the view of migrations and the spread of animals, with some evidence pointing to an introduction of this lineage to Italy and the Balkans in Roman times [3], as opposed to the previously considered Neolithic origin. However, this is still not a settled issue. Alternatively, the apparently indicine admixture could instead come from interbreeding with local aurochs. As the subspecies that gave rise to taurine and indicine cattle have separated relatively recently in evolutionary terms (about 250,000–300,000 years ago), the lineage sorting between the two might have been incomplete. In such a scenario, some European aurochs could still possess the same genes that are common in the Asian subspecies and transfer them onto the purely taurine Neolithic cattle. Indeed, this phenomenon has already been described [78]. The latter scenario is perhaps less likely than a more recent migration and breeding of Asian cattle; it cannot, however, be ruled out completely [3].

Y-chromosome variation of modern breeds reveals that all Podolian breeds carry some form of the Y2 haplogroup [54]. This is shared by some other Central European breeds, but the specific variants differ.

### 4.3. Hungarian Grey Cattle

The diversity of European breeds has never reached the diversity observed, for example, in Africa. But some of the breeds known in Central Europe are of particular interest. In the Neolithic, the longhorn type of domestic cattle (sometimes called *primigenius* type) was common and spread. This changed during the times, and in the Central European Middle Ages, the typical form were cattle with short, sometimes very small horns (called *brachyceros*) [79]. Nevertheless, even in this area long-horn cattle persists in the form of Hungarian Gray cattle. The long horns are a well-known and marked feature of regional breeding [80]. The origin of this breed was debated, and even the idea that these cattle descended directly from aurochs (*Bos primigenius*) was raised. However, late aurochs’ contribution to this breed does not correspond to both the archaeozoological and historical records. An explicit reference to long-horned cattle, ‘*magnus cornuotes boves Hungaricos*’, first appears only in a 16th century document [80]. Genetic information currently shows some influence of the local aurochs, but this is common for a larger group of the Podolian breeds, and is not unique to the Hungarian Grey [3]. This further supports the interpretation that the hybridization occurred only in prehistory, and the current state of the breeds reflects the evolution and diversification of local domestic cattle in the last several centuries.

### 4.4. Other Historical Cattle Breeds of Central Europe

Other breeds that have been studied comprise those found in Germany and Austria and the alpine region. Of these, an interesting pattern emerges: the Fleckvieh, Murbodner, and Franken Gelbvieh cluster together and with French Tarentaise breed. Other breeds, such as Tyrolean Grauvieh and the Original Braunvieh of Murnau-Werdenfelser cluster are closer to the more eastern breeds, such as various subgroups of Busha cattle [81]. Thus, it could be concluded that some level of geneflow happened in the past between the regions of Germany and Austria, in which these breeds originate, and the Balkans, where Busha were traditionally raised. However, it might also be a result of much more recent history, as can be seen in the already mentioned Murbodner breed. It was found to be surprisingly related to the somewhat geographically distant Franken Gelbvieh. Upon further investigation, it is revealed that the latter was in fact used massively after the Second World War to improve the milk yield of the Murbodner [81]. According to the authors of the cited study, this has, in combination with subsequent selection on the introgressed genes, led to the effective loss of the genetic identity of this breed. It appears that all the breeds studied so far, mainly those found in the western half of Central Europe, form a closely related group with possible limited contact with other areas of an uncertain date.

## 5. Challenges of Genomic Approaches

When studying domestication, we should recall changes that can distinguish the general biological and genetic processes in the domestic population from the wild state [82,83,84,85]. Inbreeding, quick genetic drift, founder events, and the bottleneck effect are typical phenomena which can affect small herds originating from capturing wild animals and their domestication, but also arising during the spatial expansion of the farmers; such pioneering actions put the herds in areas where a low possibility of crossing took place. The domestic herds lost contact with the source of diversity in the area of domestication. The lowering of genetic diversity, along the distribution routes from the origin of domestication, migration, or export, is another consequence. Within the artificial selection, a common breeding practice is castration, possibly leading to the elimination of some phenotypes and further decreasing diversity. Early mating, which has been described in some domesticated species [86], would lead to a shortening of the generation rate. Other phenomena compose the so called “domestication syndrome”, known already to C. Darwin and so far intensively investigated, causing appearance of more particular phenotypic traits at once [86], and “the cost of domestication” [87]. Such processes can affect both the arranging and interpretation of genetical research and its linking to phenotypic manifestation. However, there is no space to analyse all general processes here; rather, we point to some concrete possibilities.

The methods of mitochondrial DNA analysis, usually restricted to the D-loop variable region, and Y chromosome haplogroups to document the history of the formation of modern cattle are approaching their limits as far as comprehensive data for the European region have been collected. Partial progress has been achieved by the extension of the sequenced region in ancient DNA to a complete mitochondrial DNA. This advancement can be documented by the publication of complete mitochondrial genome of aurochs [88]. The efforts to apply the polymorphism in additional specific genes, like the cytochrome c gene [89], or to employ the microsatellite sequences throughout the bovine genome have also had only a limited impact.

The prospects of the application genomics methods to the elucidation of the domestic species origin have been summarized by Frantz et al. [90].

Nevertheless, the spread of new generation sequencing in archaeogenetics to extend the studied region will always be hampered by the degree of DNA fragmentation in ancient samples. This process restricts the size of amplified and sequenced fragments even in the limited percent of archaeological finds still containing DNA under the environmental conditions of Central Europe [91]. The inevitable degradation also prevents the transfer of new technologies of long-range sequencing to the ancient samples, although this progress greatly simplifies targeted resequencing in extant populations. This can be documented by a full mtDNA survey in a range of cattle breeds across Central Europe and related regions obtained by long-range sequencing [43].

Therefore, the sequencing of a complete genome of aurochs [78] represent an important milestone in archaeogenetics of cattle [92]. This work firstly demonstrated the feasibility of this approach in the historical and pre-historical finds of aurochs and cattle.

In extant cattle, the whole-genome sequencing became a standard approach after the publication of a cattle reference genome [93]. However, the reference genome was derived from an animal belonging to the Hereford breed, which is genetically quite distant from the Central European historical breeds [81]. Later on, breed-specific whole-genome sequences were obtained for the main commercial cattle breeds. If not prevented by commercial reasons or the rules of still unaccomplished projects, the breed-specific sequences are available in public databases, e.g., on the server run by the University of Missouri (http://bovinemine-v16.rnet.missouri.edu/bovinemine/begin.do, accessed on 15 February 2024).

On the other hand, the collecting of genomic information for historical breeds, after the initial delay due to their low economic impact, is progressing at a high speed. For example, the genome of more than 300 animals have been sequenced in the German Black Pied breed, an endangered historical breed [94]. Its initial farming dates back to the 18th century in the North Sea region of Germany and the Netherlands (as Dutch Friesian). This breed also gave rise to the modern Holstein breed.

Similarly, the full genome sequence of the Czech Red cattle was already sequenced in February 2018. This result was the first fully read animal genome in the Czech Republic, including humans [72]. Although approximately 12.5 million single nucleotide polymorphisms have been found distinguishing this breed from the reference sequence of the Hereford breed animal, the specific features still have to be interpreted.

The gene pool of the existing herd of Czech Red Pied cattle was also sequenced in 2018 [62,95]. Since the breed of Simmental group probably originated by crossing the locally adapted breeds with imported Simmental animals, it is assumed that a significant part of the original genetic information might be contained in the gene pool of the contemporary population. Moreover, the original gene pool is preserved in the frame of the nucleus herd of approximately 30 animals established in 2010 and reflecting the state of the breed in the 1990s (https://vuzv.cz/en/genetic-resources/, accessed on 15 February 2024), and is available for comparative studies.

The relatedness of populations of historical breeds as well as ancient finds can be based simply on low coverage whole-genome sequencing, without using specific targeted genes [72]. However, the future development is delineated by new approaches published. For example, the archaeogenomic group around University College in Dublin succeeded in the identification of 1620 genes serving as micro-RNA receptors that have been modified in the course of domestic cattle evolution from the wild aurochs [96]. These receptor sequences perform a role in the regulation of genes. Consequently, this paper provides a network of polymorphisms that can be exploited for tracking the individual steps in the transition from aurochs to domestic cattle.

Finally, the combination of genomic and archaeogenetic methods can provide the necessary data to build breeding plans for rare animal genetic resources, including the next efforts of restoration of the extinct aurochs by so-called back-breeding (https://breedingback.blogspot.com, accessed on 15 December 2023).

## 6. Conclusions and Future Priorities

The existing studies of cattle archaic genetic material brought a significant volume of knowledge. Nevertheless, they have not yet reached the level of archaeogenomic studies performed on a horse or dog, such as in the European Research Council projects PEGASUS or AGRICON; see [97,98]. A large number of genetic, archaeogenetic, and archaeogenomic publications evaluate and interpret molecular data alone, independently of other kinds of evidence. In summary, we would like to stress the importance of combining different kinds of information and different approaches related to each investigated archaeological find, such as morphological data, archaeological context, and molecular data. The appropriateness of such an approach is illustrated in some of the mentioned studies as well as in the text itself. This multidisciplinary approach includes confronting the genetic and phenotypic aspect of the observed state, combining knowledge of haplotype, sex, size, and status of the individual, along with other informative traits.

## Data Availability

No new data were created or analyzed in this study. Data sharing is not applicable to this article.

## References

[1] Sherratt A., Hodder I., Isaac G., Hammond N. (1981). Plough and pastoralism: Aspects of the secondary products revolution. Pattern of the Past: Studies in Honour of David Clarke.

[2] Sherratt A. (1983). The secondary exploitation of animals in the old world. World Archaeol..

[3] Senczuk G., Mastrangelo S., Ajmone-Marsan P., Becskei Z., Colangelo P., Colli L., Ferretti L., Karsli T., Lancioni H., Lasagna E. (2021). On the origin and diversification of Podolian cattle breeds: Testing scenarios of European colonization using genome-wide SNP data. Genet. Sel. Evol..

[4] Achilli A., Bonfiglio S., Olivieri A., Malusà A., Pala M., Kashani B.H., Perego U.A., Ajmone-Marsan P., Liotta L., Semino O. (2009). The multifaceted origin of taurine cattle reflected by the mitochondrial genome. PLoS ONE.

[5] Helmer D., Gourichon L., Monchot H., Peters J., Saña Segui M., Vigne J.-D., Peters J., Helmer D. (2005). Identifying early domestic cattle from prepottery Neolithic sites on the middle Euphrates using sexual dimorphism. The First Steps of Animal Domestication.

[6] Troy C.S., MacHugh D.E., Bailey J.F., Magee D.A., Loftus R.T., Cunningham P., Chamberlain A.T., Sykes B.C., Bradley D.G. (2001). Genetic evidence for Near-Eastern origins of European cattle. Nature.

[7] Edwards C.J., Bollongino R., Scheu A., Chamberlain A., Tresset A., Vigne J.D., Baird J.F. (2007). Mitochondrial DNA analysis shows a Near Eastern Neolithic origin for domestic cattle and no indication of domestication of European aurochs. Proc. R. Soc. B Biol. Sci..

[8] Zeder M.A. (2008). Domestication and early agriculture in the Mediterranean Basin: Origins, diffusion, and impact. Proc. Natl. Acad. Sci. USA.

[9] Bollongino R., Burger J., Powell A., Mashkour M., Vigne J.D., Thomas M.G. (2012). Modern taurine cattle descended from small number of near-Eastern founders. Mol. Biol. Evol..

[10] Scheu A., Powell A., Bollongino R., Vigne J.-D., Tresset A., Çakırlar C., Benecke N., Burger J. (2015). The genetic prehistory of domesticated cattle from their origin to the spread across Europe. BMC Genet..

[11] Verdugo M.P., Mullin V.E., Scheu A., Mattiangeli V., Daly K.G., Delser P.M., Hare A.J., Burger J., Collins M.J., Kehati R. (2019). Ancient cattle genomics, origins, and rapid turnover in the Fertile Crescent. Science.

[12] Scheu A., Hartz S., Schmölcke U., Tresset A., Burger J., Bollongino R. (2008). Ancient DNA provides no evidence for independent domestication of cattle in Mesolithic Rosenhof, Northern Germany. J. Archaeol. Sci..

[13] Beja-Pereira A., Caramelli D., Lalueza-Fox C., Vernesi C., Ferrand N., Casoli A., Goyache F., Royo L.J., Conti S., Lari M. (2006). The origin of European cattle: Evidence from modern and ancient DNA. Proc. Natl. Acad. Sci. USA.

[14] Ginja C., Guimarães S., da Fonseca R.R., Rasteiro R., Rodríguez-Varela R., Simões L.G., Sarmento C., Belarte M.C., Kallala N., Torres J.R. (2023). Iron age genomic data from Althiburos—Tunisia renew the debate on the origins of African taurine cattle. iScience.

[15] Hristov P., Sirakova D., Mitkov I., Spassov N., Radoslavov G. (2018). Balkan brachicerous cattle—The first domesticated cattle in Europe. Mitochondrial DNA Part A.

[16] Peške L. (1994). The history of natural scientific methods in the Archaeological Institute and their present objectives. Památ. Archeol..

[17] Kovačiková L., Bréhard S., Šumberová R., Balasse M., Tresset A. (2012). New insights into the subsistence and early farming from Neolithic settlements in Central Europe: Archaeozoological evidence from the Czech Republic. Archaeofauna.

[18] Altuna J., Mariezkurrena K. (2017). Origenes y Evolucion de la Domesticacion en el Pais Basque. In European Iconography of Animals Domestics, Administración de la Comunidad Autónoma del País Vasco. https://www.euskadi.eus/contenidos/informacion/kultura_ondare_argitalpenak/es_def/adjuntos/Origenes-y-evolucion-de-la-domesticacion-en-el-Pais-Basque.-European-Iconography-of-animals-domestics.pdf.

[19] Glass M. (1991). Animal Production Systems in Neolithic Central Europe.

[20] Benecke N. (1994). Archäozoologische Studien zur Entwicklung der Haustierhaltung in Mitteleuropa und Südskandinavien von den Anfängen bis zum Ausgehenden Mittelalter.

[21] Pucher E. (2004). Der mittelneolithische Tierknochenkomplex von Melk-Winden (Niederösterreich). Ann. Naturhist. Mus. Wien.

[22] Pucher E. (2006). Ein neuer Tierknochenfundkomplex aus einer Siedlung der Badener Kultur in Ossarn bei Herzogenburg in Niederosterreich. Archäologie Osterr..

[23] Kyselý R. (2016). The size of domestic cattle, sheep, goats and pigs in the Czech Neolithic and Eneolithic Periods: Temporal variations and their causes. Archaeofauna.

[24] Altuna J., Mariezkurrena K. (2008). Animal feed remains of the inhabitants of the Arenaza cave (Basque Country) during the bronze age. Veleia.

[25] Buňátová M. (2013). Pražští Kupci na Cestách.

[26] O'Sullivan N.J., Teasdale M.D., Mattiangeli V., Maixner F., Pinhasi R., Bradley D.G., Zink A. (2016). A whole mitochondria analysis of the Tyrolean Iceman’s leather provides insights into the animal sources of Copper Age clothing. Sci. Rep..

[27] Macháček J., Nedoma R., Dresler P., Sculz I., Lagonik E., Johnson S., Kaňáková L., Slámová A., Llamas B., Wegmann D. (2021). Runes from Lany (Czech Republic)—The oldest inscription among Slavs. A new standard for multidisciplinary analysis of runic bones. J. Archaeol. Sci..

[28] Łukaszewicz K. (1952). Tur. Ochr. Przyr..

[29] Clutton-Brock J. (1999). A Natural History of Domesticated Mammals.

[30] Guintard C., Rewerski J., Bodson L. (1999). Disparition de l’Aurochs en Pologne au XVIIe sičcle, et projet de réintroduction de l’Aurochs-reconstitué en Mazury. Animaux Perdus, Animaux Retrouvés: Réapparition ou Réintroduction en Europe Occidentale D’espčces Disparues de Leur Milieu D’origine. Journée d’étude, 21 Mars 1998. Colloques D’histoire des Connaissances Zoologiques 10.

[31] Rokosz M. (1995). History of the aurochs (*Bos taurus primigenius*) in Poland. Anim. Genet. Res. Inf..

[32] van Vuure C. (2005). Retracing the Aurochs: History, Morphology and Ecology of An Extinct Wild Ox.

[33] Kyselý R. (2008). Aurochs and potential crossbreeding with domestic cattle in Central Europe in the Eneolithic period. Anthropozoologica.

[34] Kyselý R., Meduna P. (2009). O zvířeti velkém jako slon, mezi jehož rohy si mohou sednout tři muži. Pratur ve středověku Čech a Moravy—Historická a archeozoologická analýza. Památky Archeol..

[35] Bökönyi S. (1962). Zur Naturgeschichte des Ures in Ungarn und das Problem der Domestikation des Hausrindes. Acta Archaeol. Acad. Sci. Hung..

[36] Bökönyi S., Ucko P., Dembleby G. (1969). Archaeological problems and methods of recognizing animal domestication. The Domestication and Exploitation of Plants and Animals.

[37] Bökönyi S. (1974). History of Domestic Mammals in Central and Eastern Europe.

[38] Crabtree P.J. (1993). Early animal domestication in the Middle East and Europe. Archaeol. Method Theor..

[39] Bollongino R., Edwards C., Alt K., Burger J., Bradley D. (2006). Early history of European domestic cattle as revealed by ancient DNA. Biol. Lett..

[40] Bollongino R., Elsner J., Vigne J.D., Burger J. (2008). Y-SNPs do not indicate hybridisation between European aurochs and domestic cattle. PLoS ONE.

[41] Bonfiglio S., Achilli A., Olivieri A., Negrini R., Colli L., Liotta L., Ajmone-Marsan P., Torroni A., Ferretti L. (2010). The enigmatic origin of bovine mtDNA haplogroup R: Sporadic interbreeding or an independent event of Bos primigenius domestication in Italy?. PLoS ONE.

[42] Mona S., Catalano G., Lari M., Larson G., Boscato P., Casoli A., Sineo L., Di Patti C., Pecchioli E., Caramelli D. (2010). Population dynamic of the extinct European aurochs: Genetic evidence of a north-south differentiation pattern and no evidence of post-glacial expansion. BMC Evol. Biol..

[43] Cubric-Curik V., Novosel D., Brajkovic V., Stabelli O.R., Krebs S., Sölkner J., Salamon D., Ristov S., Berger B., Trivizaki S. (2022). Large-scale mitogenome sequencing reveals consecutive expansions of domestic taurine cattle and supports sporadic aurochs introgression. Evol. Appl..

[44] Anderung C., Bouwman A., Persson P., Carretero J.M., Ortega A.I., Elburg R., Smith C., Arsuaga J.L., Ellegren H., Götherström A. (2005). Prehistoric contacts over the Straits of Gibraltar indicated by genetic analysis of Iberian Bronze Age cattle. Proc. Natl. Acad. Sci. USA.

[45] Schibler J., Elsner J., Schlumbaum A. (2014). Incorporation of aurochs into a cattle herd in Neolithic Europe: Single event or breeding?. Sci. Rep..

[46] Kyselý R., Hájek M. (2012). MtDNA haplotype identification of aurochs remains originating from the Czech Republic (Central Europe). Envir. Archaeol..

[47] Wright E., Viner-Daniels S. (2015). Geographical variation in the size and shape of the European aurochs (*Bos primigenius*). J. Archaeol. Sci..

[48] Degerbøl M., Fredskild B. (1970). The Urus (Bos primigenius Bojanus) and Neolithic Domesticated Cattle (Bos Taurus Domesticus Linne) in Denmark.

[49] Lasota-Moskalewska A. (1980). Morphotic changes of domestic cattle skeleton from the Neolithic Age to the beginning of the Iron Age. Wiadomości Archeol..

[50] Müller H.H. (1964). Die Haustiere der Mittleldeutschen Bandkeramiker. Naturwissenschaftliche Beiträge Vor Frühgeschichte.

[51] Döhle H.-J. (1990). Linearbandkeramische Tierknochen von Eilsleben, Kr. Wanzleben—Einige Aspekte der frühen Haustierhaltung. Jahresschr. Mitteldtsch. Vorgesch..

[52] Bogucki P.I. (1989). Pre-urban settlements in prehistoric temperature Europe. Am. J. Archaeol..

[53] Götherström A., Anderung C., Hellborg L., Elburg R., Smith C., Bradley D.G., Ellegren H. (2005). Cattle domestication in the Near East was followed by hybridization with aurochs bulls in Europe. Proc. Royal Soc. B Biol. Sci..

[54] Edwards C.J., Ginja C., Kantanen J., Pérez-Pardal L., Tresset A., Stock F., Gama L.T., Penedo M.C.T., Bradley D.G., Lenstra J.A. (2011). et al. Dual origins of dairy cattle farming—Evidence from a comprehensive survey of european Y-chromosomal variation. PLoS ONE.

[55] Wang S.L., Nan Z.R., Prete D. (2016). Protecting wild yak (*Bos mutus*) species and preventing its hybrid in China. J. Arid Land.

[56] Fontanesi L., Tazzoli M., Russo V., Beever J. (2010). Genetic heterogeneity at the bovine *KIT* gene in cattle breeds carrying different putative alleles at the spotting locus. Anim. Genet..

[57] Medugorac I., Seichter D., Graf A., Russ I., Blum H., Göpel K.H., Rothammer S., Förster M., Krebs S. (2012). Bovine polledness--an autosomal dominant trait with allelic heterogeneity. PLoS ONE.

[58] Kasprzak-Filipek K., Sawicka-Zugaj W., Litwińczuk Z., Chabuz W., Šveistienė R., Bulla J. (2020). Polymorphism of the melanocortin 1 receptor (*MC1R*) gene and its role in determining the coat colour of Central European cattle breeds. Animals.

[59] Novák K., Pikousová J., Czerneková V., Mátlová V. (2017). Diversity of the TLR4 immunity receptor in Czech native cattle breeds revealed using the Pacific Biosciences sequencing platform. Anim. Biotech..

[60] Telldahl Y., Svensson E., Götherström A., Storå J. (2011). Typing late prehistoric cows and bulls-osteology and genetics of cattle at the Eketorp Ringfort on the Oland Island in Sweden. PLoS ONE.

[61] Hořín P., Vojtíšek P., Vyskočil M., Majzlík I. (1997). Major histocompatibility complex class I (BoLA-A) polymorphism in Czech red cattle. Živočišná Výrob..

[62] Novák K., Bjelka M., Samake K., Valčíková T. (2019). Potential of TLR-gene diversity in Czech indigenous cattle for resistance breeding as revealed by hybrid sequencing. Arch. Anim. Breed..

[63] Felius M., Beerling M.L., Buchanan D.S., Theunissen B., Koolmees P.A., Lenstra J.A. (2014). On the History of Cattle Genetic Resources. Diversity.

[64] Taberlet P., Valentini A., Rezaei H.R., Naderi S., Pompanon F., Negrini R., Ajmone-Marsan P. (2008). Are cattle, sheep, and goats endangered species?. Mol. Ecol..

[65] Upadhyay M.R., Chen W., Lenstra J.A., Goderie C.R.J., MacHugh D.E., Park S.D.E., Magee D.A., Matassino D., Ciani F., European Cattle Genetic Diversity Consortium (2017). Genetic origin, admixture and population history of aurochs (*Bos primigenius*) and primitive European cattle. Heredity.

[66] Sørensen A.C., Sørensen M.K., Berg P. (2005). Inbreeding in Danish dairy cattle breeds. J. Dairy Sci..

[67] Ludwig A., Lieckfeldt D., Hesse U.G.W., Froelich K. (2016). Tracing the maternal roots of the domestic Red Mountain Cattle. Mitochondrial DNA.

[68] Szarek J., Adamczyk K., Felenczak A. (2004). Polish Red Cattle breeding: Past and present. Anim. Genet. Res. Inf..

[69] Czerneková V., Kott T., Dudková G., Sztankóová Z., Soldát J. (2006). Genetic diversity between seven Central European cattle breeds as revealed by microsatellite analysis. Czech J. Anim. Sci..

[70] Čítek J., Panicke L., Řehout V., Procházková H. (2006). Study of genetic distances between cattle breeds of Central Europe. Czech J. Anim. Sci..

[71] Kasprzak-Filipek K., Sawicka-Zugaj W., Litwificzuk Z., Chabuz W., Sveistiene R., Bulla J. (2019). Assessment of the genetic structure of Central European cattle breeds based on functional gene polymorphism. Global Ecol. Conserv..

[72] Novák K., Kyselová J., Czerneková V., Mátlová V. (2018). Genomic data as a prerequisite for efficient conservation programme of the Czech Red cattle. Book of Abstracts of the 69th Annual Meeting of the European Federation of Animal.

[73] Čítek J., Košvanec K., Řehout V., Hajič F., Šoch M. (1997). Czech Red cattle—An endangered genetic line (in Slovak). Polnohospodárstvo.

[74] Petrakova L., Kerziene S., Razmaite V. (2012). Contribution of different breeds to lithuanian red cattle using pedigree information with only a fraction of the population analyzed. Vet. Ir Zootech..

[75] LDi Lorenzo P., Lancioni H., Ceccobelli S., Colli L., Cardinali I., Karsli T., Capodiferro M.R., Sahin E., Ferretti L., Marsan P.A. (2018). Mitochondrial DNA variants of Podolian cattle breeds testify for a dual maternal origin. PLoS ONE.

[76] Mastrangelo S., Tolone M., Ben Jemaa S., Sottile G., Di Gerlando R., Cortés O., Senczuk G., Portolano B., Pilla F., Ciani E. (2020). Refining the genetic structure and relationships of European cattle breeds through meta-analysis of worldwide genomic SNP data, focusing on Italian cattle. Sci. Rep..

[77] Decker J.E., McKay S.D., Rolf M.M., Kim J., Alcalá A.M., Sonstegard T.S., Hanotte O., Götherström A., Seabury C.M., Praharani L. (2014). Worldwide patterns of ancestry, divergence, and admixture in domesticated cattle. PLoS Genet..

[78] Park S.D.E., Magee D.A., McGettigan P.A., Teasdale M.D., Edwards C.J., Lohan A.J., Murphy A., Braud M., Donoghue M.T., Liu Y. (2015). Genome sequencing of the extinct Eurasian wild aurochs, *Bos primigenius*, illuminates the phylogeography and evolution of cattle. Genome Biol..

[79] Bökönyi S. (1971). The development and history of domestic animals in Hungary: The neolithic through the Middle Ages. Amer. Anthropol..

[80] Bartosiewicz L. (1997). The Hungarian Grey cattle: A traditional European breed. Anim. Genet. Resour./Resour. Génét. Anim./Recur. Genét. Anim..

[81] Medugorac I., Medugorac A., Russ I., Veit-Kensch C.E., Taberlet P., Luntz B., Mix H.M., Förster M. (2009). Genetic diversity of European cattle breeds highlights the conservation value of traditional unselected breeds with high effective population size. Mol. Ecol..

[82] Zeder M.A. (2012). Pathways to Animal Domestication.

[83] Zeder M.A. (2015). Core questions in domestication research. Proc. Natl. Acad. Sci. USA.

[84] Hunter P. (2018). The genetics of domestication: Research into the domestication of livestock and companion animals sheds light both on their “evolution” and human history. EMBO Rep..

[85] Ahmad H.I., Ahmad M.J., Jabbir F., Ahmr S., Ahmad N., Elokil A.A., Ch J. (2020). The domestication makeup: Evolution, survival, and challenges. Front. Ecol. Evol. Sec. Evol. Pop. Genet..

[86] Wilkins A.S., Wrangham R.W., Fitch W.T. (2014). The “Domestication Syndrome” in mammals: A unified explanation based on neural crest cell behavior and genetics. Genetics.

[87] Moyers B.T., Morrell P.L., McKay J.K. (2018). Genetic costs of domestication and improvement. J. Hered..

[88] Edwards C.J., Magee D.A., Park S.D.E., McGettigan P.A., Lohan A.J., Murphy A., Finlay E.K., Shapiro B., Chamberlain A.T., Richards M.B. (2010). A complete mitochondrial genome sequence from a mesolithic wild aurochs (*Bos primigenius*). PLoS ONE.

[89] Stock F., Edwards C.J., Bollongino R., Finlay E.K., Burger J., Bradley D.G. (2009). Cytochrome b sequences of ancient cattle and wild ox support phylogenetic complexity in the ancient and modern bovine populations. Anim. Genet..

[90] Frantz L.A.F., Bradles D.G., Larson G., Orlando L. (2020). Animal domestication in the era of ancient genomics. Nat. Rev. Genet..

[91] Svensson E.M., Häsler S., Nussbaumer M., Rehazek A., Omrak A., Götherström A. (2014). Mediaeval cattle from Bern (Switzerland): An archaeozoological, genetic and historical approach. Schweiz. Arch. Tierheilkd..

[92] Sinding M.-H.S., Gilbert M.T.P. (2016). The draft genome of extinct European aurochs and its implications for de-extinction. Open Quat..

[93] Elsik C.G., Tellam R.L., Worley K.C., Gibbs R.A., Abatepaulo A.R.R., Abbey C.A., Adelson D.L., Aerts J., Ahola V., Alexander L. (2009). The genome sequence of taurine cattle. Science.

[94] Neumann G.B., Korkuc P., Arends D., Wolf M.J., May K., König S., Brockmann G.A. (2023). Genomic diversity and relationship analyses of endangered German Black Pied cattle (DSN) to 68 other taurine breeds based on whole-genome sequencing. Front. Genet..

[95] Novák K., Kyselová J., Czerneková V., Hofmannová M., Valčíková T., Samake K., Bjelka M. (2018). Diversity of innate immunity genes in the Czech Simmental cattle. Book of Abstracts of the 69th Annual Meeting of the European Federation of Animal Science.

[96] Braud M., Magee D.A., Park S.D.E., Sonstegard T.S., Waters S.M., MacHugh D.E., Spillane C. (2017). Genome-wide microRNA binding site variation between extinct wild aurochs and modern cattle identifies candidate microRNA-regulated domestication genes. Front. Genet..

[97] Librado P., Khan N., Fages A., Kusliy M.A., Suchan T., Tonasso-Calvière L., Schiavinato S., Alio glu D., Fromentier A., Perdereau A. (2021). The origins and spread of domestic horses from the Western Eurasian steppes. Nature.

[98] Bergström A., Frantz L., Schmidt R., Ersmark E., Lebrasseur O., Girdland-Flink L., Lin A.T., Storå J., Sjögren K.G., Anthony D. (2020). Origins and genetic legacy of prehistoric dogs. Science.

